# Supplementary material for: Protected quantum gates using qubit doublons in dynamical optical lattices
Source: Nature. 2026 Apr 8;652(8110):609–14. doi: 10.1038/s41586-026-10285-1 (PMC13668714; doi:10.1038/s41586-026-10285-1)
Supplement: Supplementary file 1 [file 41586_2026_10285_MOESM1_ESM.pdf]

nature portfolio

Peer Review File

Protected quantum gates using qubit doublons in dynamical optical lattices

Corresponding Author: Dr Konrad Viebahn

Any redactions in this file are there to maintain patient confidentiality, the confidentiality of unpublished data, or to remove third-party material.

This file contains all reviewer reports in order by version, followed by all author rebuttals in order by version.

Version 0:

Reviewer comments:

Referee #1

(Remarks to the Author)
Kiefer and co-authors demonstrate with neutral atoms in an optical lattice - a geometric SWAP in the non-interacting regime, and a spin-exchange entangling gate using a Feshbach-tunable interaction and a dynamical phase. To my knowledge the geometric SWAP and the particular protocol for a direct exchange gate in a double well has not been realized with neutral atoms. For the direct exchange gate, in the end what is realize is spin entanglement, and is not unique to Fermi statistics, and as such this gate competes against mechanisms such as – Rydberg entangling gates, other methods for achieving spin entanglement using contact interactions, etc. While compared clearly to the stability of a superexchange gate, the comparison points could be broader. In the context of proposals and implementation of geometric gates harnessing symmetries, I am uncertain that the full range of quantum dot literature is addressed.

Quantum dot literature: In terms of the novelty of using geometric gates with either atoms or electrons in quantum dots, the quantum dot literature continues to evolve with discussion of geometric protocols. What is the relation between the current work and for example: “High-fidelity geometric quantum gates exceeding 99.9% in germanium quantum dots, Nature Communications volume 16, 7953 (2025)” or “Single-spin-qubit geometric gate in a silicon quantum dot, Physical Review Applied 21, 014044 (2024).

Discussion of statistics and symmetry: As the authors discuss in the Methods, the fundamental idea of the dark states in this work applies equally to bosons as fermions. The symmetry is just flipped in the analysis. Although there are technical considerations regarding dealing with interactions for bosons. On this topic, I wonder about some of the phrasing in the introduction paragraph: For example “fundamental symmetries and quantum statistics into a powerful resource for fault-tolerant computation”. Why say both fundamental symmetries and quantum statistics? Is there a distinction being made? And if so is it appropriate to implicate both in the physics of this work?

Geometric SWAP (Figs. 2,3): As a presentation point, some of the key information presented about the motivation is in the Conclusion section. In this spirit, the comparison point to make would be to SWAP realizations that can be achieved with neutral atoms with concatenated two-qubit gates. How would the fidelities compare to other QC realizations with neutral atoms?

In this section the sentence “Additionally, a total acquired phase of $\varphi = \pi/2$ corresponds to an entangling √swap gate.” Either I am misunderstanding this statement or the authors should be careful of this phrasing in non-interacting case, in terms what degree of freedom is stated to be entangled.

Finite U exchange data: I am convinced of the improvement of fidelity compared to superexchange when introducing tunneling noise. However, I would also compare to spin-exchange in a single well that has been studied in a variety of contexts since the work of Anderlini et al (Ref. [12]), and provide more reasoning behind the need not to work in a single well (I think there are some). One could ask, for example, would single-well exchange be equally insensitive to lattice fluctuations, especially if adiabatic passage state preparation (which is certainly not a geometric effect, but an example of other ways to avoid instabilities) is carried out such as in Ref. [13]. It is also relevant to compare the achieved spin

entanglement to Rydberg entangling gates, and prospects for improved fidelity of the results here.

Preparation stage: Through the checkerboard lattice state preparation – 70 percent of atoms are found in a singlet state. The authors should comment on how this preparation stage still depends on lattice fluctuations, and its role in large-scale protocols that the gates in this work will ultimately be involved in.

Presentation comments: Doublon is introduced rapidly within the text, and for a general audience there might need to be more context for this term.

Referee #2

(Remarks to the Author)
In this manuscript, Kiefer et al. describe and experimentally demonstrate a two-qubit swap gate, and partial swap gate, using fermionic atoms in an optical superlattice. Fermion pairs are prepared in specific spin state on a double well, and a ramp of the energy offset realizes a controlled swap gate. A major advantage is that the acquired phase is geometric in origin, making it robust against e.g. noise in experimental parameters, and this results in a correspondingly rather high fidelity. By working at a non-zero interaction strength, a dynamical phase is also introduced which is controllable via the interaction strength.

I find this paper quite wonderful, combining both beautiful physics ideas and a very impressive experimental achievement. The quality of the experimental work and analysis/interpretation is excellent. It illustrates the advantage of exploiting geometric phases in optical lattice platforms for robust operations, and these ideas are applicable to other quantum information platforms also. I believe the work will be highly impactful on the emerging field of quantum computation in lattice-based platforms, and be a landmark paper in the literature. In particular, the direct comparison of collisional phases arising from either direct exchange or superexchange, and the higher fidelity of the former approach.

There are a couple of comments which I would like to see addressed or expanded upon. If these are satisfactorily addressed I would be happy to recommend for publication and .

1) One major comment regards state preparation. The tagline fidelity seems very impressive - 99.91% as quoted in the abstract. As I understand it, this is the fidelity of the gate itself assuming a pair is prepared in the desired initial spin state, and hence allowing one to benefit from the different evolutions of singet and triplet manifolds. However, I gather that the state preparation is only 60-70%. Therefore this seems to present the overwhelming bottleneck in overall fidelity. Can the authors comment on this limitation, and what sets/limits the state preparation fidelity?

2) Robustness of the gate against tunneling noise is briefly demonstrated in Fig 3. It is clear that the bandwidth of the noise is important - DC variation in t is already present across the lattice owing to beam inhomogeneity and is irrelevant, and as the authors indirectly note, higher bandwidth noise would lead to heating. To what extent then is the 5% robustness figure given frequency dependent? One might wonder how wide the robustness window is, and how this number depends on frequency. Furthermore, do the authors have an idea of the mechanism for falling fidelity? A 5% noise limit could actually be viewed as quite small, seeing as this gate is supposedly geometrically protected, and it's not clear what is actually breaking down when tunneling noise is introduced.

3) A small point, but one which initially confused me. In Fig 1, 1a shows an initial singlet fraction of 60%. However, in 1d the singlet fraction instead reaches a maximum of 70%. I understand that these are different data runs, and in the text the authors indeed quote a preparation fidelity of 60-70%. Is this just due to day-to-day variations? Initially it seemed that something about the different protocol in 1d led to higher singlet fractions, but it seems more likely that this is simply the fidelity of initial preparation. Is that right? Furthermore, the diagram in Fig1 seems to show that atoms are topologically pumped in different directions within the same lattice. How is this achieved, since as far as I understand all controls are global?

4) I expect that there is an adiabaticity criterion here, in that the gate must be performed slowly such that pairs remain in the dark state. What sets this timescale; I assume t? And how fine-tuned is the timescale used experimentally, i.e. how robust is the fidelity against this choice?

These are mainly small points and clarifications which I feel it would be helpful to address, and perhaps clarify in the text. Should this be done I'd be happy to recommend this paper, and congratulate the authors on the very nice work.

Version 1:

Reviewer comments:

Referee #1

(Remarks to the Author)
I thank the authors for the especially clear responses to my queries and associated changes to the manuscript.

There are a few points the authors could consider regarding the comparative descriptions to Rydberg systems:

1) The authors indicate "…, where the finite lifetime of the intermediate state poses a constraint." It does pose a constraint, but this is an oversimplification of the error budgets for Rydberg gates.
2) The authors do not write about SPAM in the context of their comparison between their system and Rydberg atoms. In the section discussing the 60-70 percent preparation efficiency, there is not a comparative discussion. While the recent work of Greiner at all realizing 99% preparation efficiency (on the arxiv) is spectacular, there are many reasons for continued uncertainty regarding quantum computing techniques requiring full very low entropy preparation of both effective spin and charge degrees of freedom.

With the above considerations, I am happy to recommend this work for publication in Nature.

Referee #2

(Remarks to the Author)
I thank the authors for their careful and conscientious response to the comments, both from me and the other referee. In particular the extra discussion of noise bandwidth/frequency dependence is much improved. I feel that the paper is in much better shape, and the confusions/questions that I had regarding state preparation, topological pumping, and gate robustness, have been satisfactorily addressed. I recommend the paper for publication, and congratulate the authors on a beautiful experiment.

Open Access This Peer Review File is licensed under a Creative Commons Attribution 4.0 International License, which permits use, sharing, adaptation, distribution and reproduction in any medium or format, as long as you give appropriate credit to the original author(s) and the source, provide a link to the Creative Commons license, and indicate if changes were made.
In cases where reviewers are anonymous, credit should be given to 'Anonymous Referee' and the source.
The images or other third party material in this Peer Review File are included in the article's Creative Commons license, unless indicated otherwise in a credit line to the material. If material is not included in the article's Creative Commons license and your intended use is not permitted by statutory regulation or exceeds the permitted use, you will need to obtain permission directly from the copyright holder.
To view a copy of this license, visit https://creativecommons.org/licenses/by/4.0/

Referee #1 (Remarks to the Author):

Kiefer and co-authors demonstrate with neutral atoms in an optical lattice - a geometric SWAP in the non-interacting regime, and a spin-exchange entangling gate using a Feshbach-tunable interaction and a dynamical phase. To my knowledge the geometric SWAP and the particular protocol for a direct exchange gate in a double well has not been realized with neutral atoms. For the direct exchange gate, in the end what is realize is spin entanglement, and is not unique to Fermi statistics, and as such this gate competes against mechanisms such as – Rydberg entangling gates, other methods for achieving spin entanglement using contact interactions, etc. While compared clearly to the stability of a superexchange gate, the comparison points could be broader. In the context of proposals and implementation of geometric gates harnessing symmetries, I am uncertain that the full range of quantum dot literature is addressed.

We would like to thank the referee for their critical reading of the manuscript and constructive feedback, in particular regarding putting our SWAP implementation on a broader footing and comparing it to other platforms.

As suggested by referee #1, we added a discussion of the state-of-the-art in Rydberg atoms to the main text (new references [41-45]) with a direct comparison to Rydberg gate fidelities. In addition, we outline strategies to increase the SWAP and exchange gate fidelities even further (Methods section I.).

Quantum dot literature: In terms of the novelty of using geometric gates with either atoms or electrons in quantum dots, the quantum dot literature continues to evolve with discussion of geometric protocols. What is the relation between the current work and for example: “High-fidelity geometric quantum gates exceeding 99.9% in germanium quantum dots, Nature Communications volume 16, 7953 (2025)” or “Single-spin-qubit geometric gate in a silicon quantum dot, Physical Review Applied 21, 014044 (2024).

The paper mentioned by the referee, in particular Zhou et al. Nature Communications (2025), marks an impressive advancement in the fidelity of single-qubit geometric gates, using germanium quantum dots. While their work is conceptually related through the use of geometric phases for quantum gates, there are also key differences which sets our work clearly apart from theirs. First, Zhou et al. demonstrate single-qubit geometric gates, compared to our two-qubit gates. Second, the geometric protocol from Zhou et al. relies on non-adiabatic pulse sequences to cancel dynamical phases, while also picking up geometric contributions. Conversely, the geometric aspect in our gate operation is completely intrinsic and dynamical phases are absent by construction.

We added the Nature Communications (2025) to our references [29].

Discussion of statistics and symmetry: As the authors discuss in the Methods, the fundamental idea of the dark states in this work applies equally to bosons as fermions. The symmetry is just flipped in the analysis. Although there are technical considerations regarding dealing with interactions for bosons. On this topic, I wonder about some of the phrasing in the introduction paragraph: For example “fundamental symmetries and quantum statistics into a powerful resource for fault-tolerant computation”. Why say both fundamental symmetries and quantum statistics? Is there a distinction being made? And if so is it appropriate to implicate both in the physics of this work?

Indeed, the separate mention of “fundamental symmetries and quantum statistics” may appear somewhat redundant. We changed the sentence to “[…] fundamental symmetries, including quantum statistics, […]”.

As pointed out by the referee, it is crucial to operate with quantum particles *with definite quantum statistics*, either bosonic or fermionic. Our separate mention of “quantum statistics” in the introduction is motivated by the fact that our scheme would not work (or only with considerable modifications) if thermal atoms were involved. Thermal atoms, whose motional degree of freedom is not totally controlled (as in most tweezer atom arrays), effectively obey classical Boltzmann statistics for distinguishable particles.

The mention of “fundamental symmetries” goes beyond quantum statistics and, in particular, refers to the chiral symmetry of the Hamiltonian (Eq. 3 for $U = 0$) and time-reversal symmetry (Methods).

Related to the referee’s comment, we added a half-paragraph to the main text introduction in which the role quantum statistics is explained in more detail (in contrast to thermal qubits for which quantum statistics is less important).

Geometric SWAP (Figs. 2,3): As a presentation point, some of the key information presented about the motivation is in the Conclusion section. In this spirit, the comparison point to make would be to SWAP realizations that can be achieved with neutral atoms with concatenated two-qubit gates. How would the fidelities compare to other QC realizations with neutral atoms?

We thank the referee for raising the important question regarding the comparison to other neutral-atom quantum computing platforms. We took up the suggestion for providing an explicit comparison of SWAP gates between our implementation and state-of-the-art Rydberg tweezer arrays, which intrinsically realise CZ-type gates. Taking into account the best published fidelities in Rydberg tweezers (99.6%) the concatenated SWAP gate (three CZs and six Hadamards) would be 98.8%. This discussion is now included in the conclusion section of the paper.

In this section the sentence “Additionally, a total acquired phase of $\phi= \pi/2$ corresponds to an entangling $\sqrt{}$swap gate.” Either I am misunderstanding this statement or the authors should be careful of this phrasing in non-interacting case, in terms what degree of freedom is stated to be entangled.

As pointed out correctly by the referee, a total phase of pi/2 is only possible in the presence of finite interactions. We have added “at finite U” and “(Section III)” to avoid any confusion.

Finite U exchange data: I am convinced of the improvement of fidelity compared to superexchange when introducing tunneling noise. However, I would also compare to spin-exchange in a single well that has been studied in a variety of contexts since the work of Anderlini et al (Ref. [12]), and provide more reasoning behind the need not to work in a single well (I think there are some). One could ask, for example, would single-well exchange be equally insensitive to lattice fluctuations, especially if adiabatic passage state preparation (which is certainly not a geometric effect, but an example of other ways to avoid instabilities) is carried out such as in Ref. [13]. It is also relevant to compare the achieved spin entanglement to Rydberg entangling gates, and prospects for improved fidelity of the results here.

We added a detailed comparison to Rydberg entangling gates to the conclusion section (see comments above) and additionally referenced specific prospects for improving our exchange gate even further (see also Methods - I).

Regarding the comparison to Anderlini et al. [12] and Kaufman et al. [13]: There are several key advantages of our scheme with respect to refs. [12-13].

Firstly, we operate in the 'drive through' regime (where the lower-energy orbital is physically displaced with respect to the higher-energy orbital), whereas refs. [12-13] follow a 'forward and backward' approach. This directly rules out any geometric phases since the backward evolution exactly cancels the forward evolution.

Secondly, our scheme readily integrates with the topological pump as scalable pathway for coupling more than just two atoms [Zhu et al. arXiv:2409.02984].

Thirdly, the schemes of refs. [12] and [13] involve auxiliary orbital-excited states of the tweezers/lattices, i.e. there are a total of three motional states involved, whereas our scheme only involves two orbitals. The excited p-orbitals in our case are shifted to much higher energies, as shown in the newly added Figure M5, and therefore do not play any role here. This directly rules out an additional source of leakage during state evolution, making the both the geometric SWAP and the direct exchange gate more robust than the existing schemes from [12-13].

Lastly, the scheme used in ref. [13] (Kaufman) utilizes two distinct orbitals of the harmonic oscillator, the resulting wavefunction overlap (between the lowest and first-excited orbital) is reduced by a factor of 2x with respect to the double-well configuration (in which the wavefunction overlap is equal to 1x Hubbard U), therefore resulting in slower gates with respect to the underlying local energy scale.

Preparation stage: Through the checkerboard lattice state preparation – 70 percent of atoms are found in a singlet state. The authors should comment on how this preparation stage still depends on lattice fluctuations, and its role in large-scale protocols that the gates in this work will ultimately be involved in.

Our current singlets preparation is limited by the finite temperature of the Fermi cloud prior to lattice loading. This could be improved by advanced trap shaping methods, such as those pioneered in [*Nature* 642, 909–915 (2025)] (Greiner group). Their state preparation results in a doublon (= singlet) preparation fidelity of 99.3(2)% and remaining 'defects' are single atoms (singlons).

In preparation for future work we explore the possibility to improving the singlet preparation fidelity even further by removing singlons from the initial state. In the figure below, we show preliminary data that uses a 'cleaning pulse' to remove three quarters of the singlons, while keeping singlets intact.

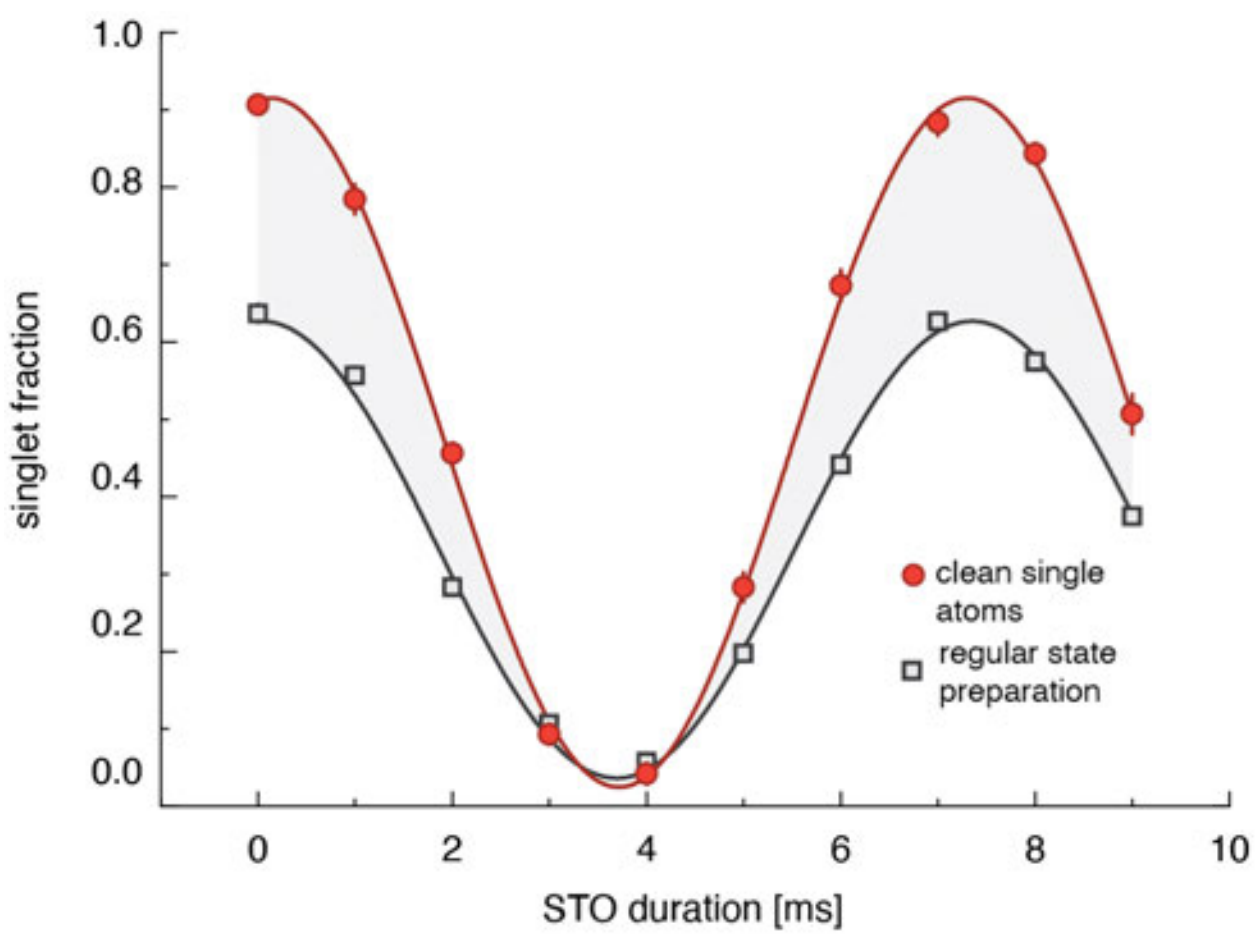

As shown in the Figure, we can already increase the singlet fraction from around 60% to above 90%, laying important groundwork for further studies with larger-scale circuits.

Presentation comments: Doublon is introduced rapidly within the text, and for a general audience there might need to be more context for this term.

In the first paragraph of the main text we included a description “Since the spatial wavefunctions of the qubits are made to overlap (qubit doublon), it is…” which hopefully adds some clarity.

Referee #2 (Remarks to the Author):

In this manuscript, Kiefer et al. describe and experimentally demonstrate a two-qubit swap gate, and partial swap gate, using fermionic atoms in an optical superlattice. Fermion pairs are prepared in specific spin state on a double well, and a ramp of the energy offset realizes a controlled swap gate. A major advantage is that the acquired phase is geometric in origin, making it robust against e.g. noise in experimental parameters, and this results in a correspondingly rather high fidelity. By working at a non-zero interaction strength, a dynamical phase is also introduced which is controllable via the interaction strength.

I find this paper quite wonderful, combining both beautiful physics ideas and a very impressive experimental achievement. The quality of the experimental work and analysis/interpretation is excellent. It illustrates the advantage of exploiting geometric phases in optical lattice platforms for robust operations, and these ideas are applicable to other quantum information platforms also. I believe the work will be highly impactful on the emerging field of quantum computation in lattice-based platforms, and be a landmark paper in the literature. In particular, the direct comparison of collisional phases arising from either direct exchange or superexchange, and the higher fidelity of the former approach.

There are a couple of comments which I would like to see addressed or expanded upon. If these are satisfactorily addressed I would be happy to recommend for publication and .

1) One major comment regards state preparation. The tagline fidelity seems very impressive - 99.91% as quoted in the abstract. As I understand it, this is the fidelity of the gate itself assuming a pair is prepared in the desired initial spin state, and hence allowing one to benefit from the different evolutions of singet and triplet manifolds. However, I gather that the state preparation is only 60-70%. Therefore this seems to present the overwhelming bottleneck in overall fidelity. Can the authors comment on this limitation, and what sets/limits the state preparation fidelity?

We thank the referee for their comment, which is also related to a question by referee #1. We have revised Method section C to clarify the details of our initial state preparation and how its efficiency relates to the fidelity of the gate.

Our protocol yields a final state where 60-70% of the atoms form paired singlets (|s⟩), while the remainder are unpaired atoms in singly occupied unit cells (singlons). Our initial preparation efficiency is limited by the starting temperature of the gas, as well as by heating that occurs during the checkerboard lattice loading procedure. However, the measured gate fidelity is fundamentally decoupled from this preparation efficiency. While finite temperature and entropy do affect the overall filling of the lattice, they do not degrade the singlet fidelity of the successfully formed atom pairs in a dimer. This is because our preparation is based on splitting doublons in the checkerboard lattice, and fermionic anti-symmetrization guarantees these pairs are in a perfect spin singlet state. As our gate protocol does not produce a measurable effect on the unpaired singlons, the measured gate fidelity is independent of the initial preparation efficiency.

We are actively working to improve our preparation efficiency (see preliminary experimental data in response to referee #1) and emphasize that this is not a fundamental limit, as fidelities exceeding 99% have already been demonstrated with a similar preparation protocol [*Nature* 642, 909–915 (2025)].

2) Robustness of the gate against tunneling noise is briefly demonstrated in Fig 3. It is clear that the bandwidth of the noise is important - DC variation in t is already present across the lattice owing to beam inhomogeneity and is irrelevant, and as the authors indirectly note, higher bandwidth noise would lead to heating. To what extent then is the 5% robustness figure given frequency dependent? One might wonder how wide the robustness window is, and how this number depends on frequency. Furthermore, do the authors have an idea of the mechanism for falling fidelity? A 5% noise limit could actually be viewed as quite small, seeing as this gate is supposedly geometrically protected, and it's not clear what is actually breaking down when tunneling noise is introduced.

We thank the referee for this insightful question regarding the frequency dependence of the noise robustness and the mechanism for the fidelity drop. We have significantly expanded the manuscript to provide a comprehensive answer, supported by new data (Fig. M5) and new analysis in both the main text and Methods Section I.

To directly address the frequency dependence, we now provide new quantitative data in Figure M5. We show that while a 2 kHz noise bandwidth allows for a 5% robustness window, increasing the bandwidth to 3 kHz shrinks this window to 3%. We have clarified in the main text that this behaviour is explained by the underlying mechanism for the fidelity drop: the geometric protection is not absolute but relies on the system evolving adiabatically within the gapped dark-state manifold. The fidelity drops when high-frequency components of the applied noise are sufficient to break this adiabaticity and drive non-adiabatic transitions. We provide a more detailed picture in Methods I, explaining that this leakage corresponds to non-adiabatic, multi-photon transitions between the lowest two Bloch bands, which are separated by a 5.6-8.1 kHz energy gap. The new figure supports this by showing both the robustness data and the calculated band structure.

3) A small point, but one which initially confused me. In Fig 1, 1a shows an initial singlet fraction of 60%. However, in 1d the singlet fraction instead reaches a maximum of 70%. I understand that these are different data runs, and in the text the authors indeed quote a preparation fidelity of 60-70%. Is this just due to day-to-day variations? Initially it seemed that something about the different protocol in 1d led to higher singlet fractions, but it seems more likely that this is simply the fidelity of initial preparation. Is that right? Furthermore, the diagram in Fig1 seems to show that atoms are topologically pumped in different directions within the same lattice. How is this achieved, since as far as I understand all controls are global?

We thank the referee for this sharp observation. We believe the referee is referring to the data presented in Figure 2. The referee's final assessment is exactly right. The difference in the measured singlet fraction between the panels reflects the typical variation in our initial state preparation fidelity, which ranges from 60-70%.

We have updated Methods Section C to clarify this point. We now explain that this variation depends on initial experimental conditions like atom number and temperature, as discussed in the reply to referee’s comment 1).

Regarding how we realize bidirectional topological pumping within the same lattice, the direction of the transport depends not only on how the lattice is modulated, but also depends on the quantum state in which the atom populates. In our superlattice, atoms prepared in the localized Wannier states of the lowest and the first excited band are transported in opposite direction due to opposite topological invariants of the respective bands. With dynamical control of the superlattice potential, we can coherently prepare one

atom from a pair to lowest band and the other to the first excited band and therefore split the pair [Zhu et al. arXiv:2409.02984]. The bidirectional transport can also be used to connect distant atoms as we illustrated in FIG. 1a. This ability to reliably move and reconnect atoms, together with the robust quantum gate we present in the current paper, creates a new toolbox for building complex quantum circuits.

4) I expect that there is an adiabaticity criterion here, in that the gate must be performed slowly such that pairs remain in the dark state. What sets this timescale; I assume t? And how fine-tuned is the timescale used experimentally, i.e. how robust is the fidelity against this choice?

The referee is correct that the gate's performance relies on meeting an adiabaticity criterion to keep the atom pairs in the dark state. We have added a detailed analysis of this to Methods Section I.

As the referee rightly assumes, the relevant timescale is set by the tunnelling energy t. Specifically, the system must evolve slowly compared to the inverse of the minimum energy gap protecting the dark state, which is approximately 2t throughout the operation. Experimentally, neither the tunnelling t nor the gate speed are fine-tuned parameters. The gate speed, in particular, is currently limited by the technical bandwidth of our superlattice phase lock. To quantify the performance, we estimate the non-adiabatic error using the Landau-Zener formula. For our experimental parameters, this yields a low excitation probability of $\sim 4 \times 10^{-7}$. This confirms the gate operates deep within the adiabatic regime, and its fidelity is therefore robust against small variations in its timing.

These are mainly small points and clarifications which I feel it would be helpful to address, and perhaps clarify in the text. Should this be done I'd be happy to recommend this paper, and congratulate the authors on the very nice work.

Dear ,

Many thanks for the second round of referee reports of our manuscript “Protected quantum gates using qubit doublons in dynamical optical lattices”.

We made two changes to the text of the Methods section in response to referee #2:

Referee comment 1) The authors indicate “…, where the finite lifetime of the intermediate state poses a constraint.” It does pose a constraint, but this is an oversimplification of the error budgets for Rydberg gates.

We changed the wording of this sentence to “…, where the finite lifetime of the intermediate state leads to decoherence.”

Referee comment 2) The authors do not write about SPAM in the context of their comparison between their system and Rydberg atoms. In the section discussing the 60-70 percent preparation efficiency, there is not a comparative discussion. While the recent work of Greiner at all realizing 99% preparation efficiency (on the arxiv) is spectacular, there are many reasons for continued uncertainty regarding quantum computing techniques requiring full very low entropy preparation of both effective spin and charge degrees of freedom.

We agree with the referee that our state preparation should be improved in order to realise a fully universal quantum computer. In this spirit, we have introduced the following sentence to the Methods: “In view to realising a fully programmable quantum computer we emphasise that our state preparation scheme should be further improved.”

[Redacted text]
